# Research Progress on Mechanism of Podocyte Depletion in Diabetic Nephropathy

**DOI:** 10.1155/2017/2615286

**Published:** 2017-07-16

**Authors:** Haoran Dai, Qingquan Liu, Baoli Liu

**Affiliations:** ^1^Department of Nephrology, Shunyi Branch, Beijing Hospital of Traditional Chinese Medicine, Station East 5, Shunyi District, Beijing 101300, China; ^2^Beijing Hospital of Traditional Chinese Medicine Affiliated to Capital Medical University, 23 Meishuguanhou Street, Dongcheng District, Beijing 100010, China

## Abstract

Diabetic nephropathy (DN) together with glomerular hyperfiltration has been implicated in the development of diabetic microangiopathy in the initial stage of diabetic diseases. Increased amounts of urinary protein in DN may be associated with functional and morphological alterations of podocyte, mainly including podocyte hypertrophy, epithelial-mesenchymal transdifferentiation (EMT), podocyte detachment, and podocyte apoptosis. Accumulating studies have revealed that disruption in multiple renal signaling pathways had been critical in the progression of these pathological damages, such as adenosine monophosphate-activated kinase signaling pathways (AMPK), wnt/*β*-catenin signaling pathways, endoplasmic reticulum stress-related signaling pathways, mammalian target of rapamycin (mTOR)/autophagy pathway, and Rho GTPases. In this review, we highlight new molecular insights underlying podocyte injury in the progression of DN, which offer new therapeutic targets to develop important renoprotective treatments for DN over the next decade.

## 1. Introduction

Diabetic nephropathy (DN), as the primary cause of end-stage renal failure, is one of the most serious complications in diabetic patients, which develops in up to 30%–40% of patients with types 1 or 2 diabetes mellitus [1]. An early sign of DN is an increased amount of urinary protein and characterized by mesangial nodular hyperplasia and thickening of the glomerular basement membrane (GBM). Microalbuminuria plays an important role in the change of the GBM. Additionally, a detailed renal biopsy data analysis regarding diabetics showed that diabetic kidney damage would include visceral epithelial cells and sertoli cell dysfunction [2, 3]. Although some researches demonstrated that podocyte injury had association with the development of DN [4], the mechanisms underlying this association are still not entirely understood and need to be further investigated.

## 2. Proteinuria and Podocyte Injury in DN

Glomerular epithelial cells, also called podocytes, are highly specialized cells, which are composed of cytoskeletal structure, joint connection, and branching foot processes circling the GBM. Podocytes, as terminal differentiation cells, are important functional cells in the glomerulus and can not regenerate when they suffer from injury. Their damage and apoptosis could result in the destruction of the glomerular filtration membrane and induce unfavorable factors in DN [5]. Foot processes are consisted of basal aspects, basolateral region and parietal region, which firmly adhere to the GBM through podoplanin protein. The basolateral region was found interdigitating with the neighboring basolateral region by slit diaphragm (SD) [6]. Furthermore, transmembrane proteins connect adapter proteins with actin cytoskeleton to maintain filtration barrier structure and function. In the podocyte parietal region, salivary proteins are used to maintain a filtration way among adjacent foot processes in the GBM through sufficient negatively charging [7]. Podocytes play an important role in pathological mechanisms underlying DN. Imasawa et al. [8] declared that structure and function in podocyte molecules transformed in high-glucose conditions, which resulted from suppression of myocyte-specific enhancer factor 2C (MEF2C) and myogenic factor 5 (MYF5) expressions through using a conditionally differentiating human podocyte cell line.

## 3. Pathomechanism of Podocyte Injury

### 3.1. Podocyte Hypertrophy

**Figure 1:**
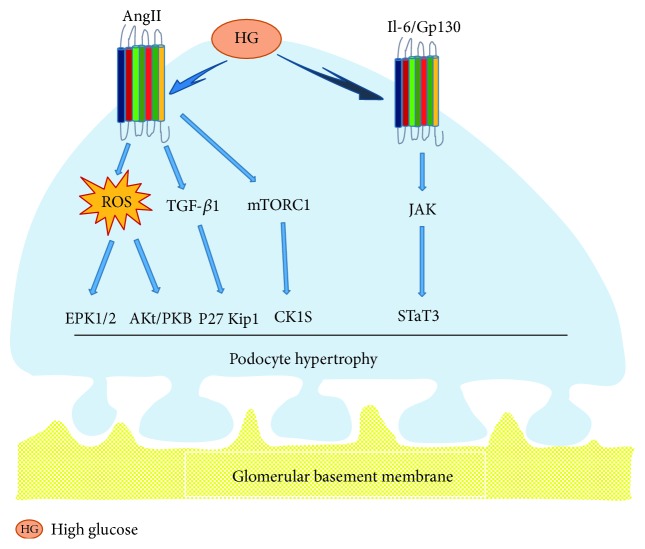
Podocyte hypertrophy. In elevated glucose levels, AngII could elevate protein expressions of kinases ERK1/2 and Akt/PKB through ROS, trigger activation of p27Kip1 via TGF-*β*1 signal pathway, or upregulate protein expression of CKIs by activating mTORC1, which eventually resulted in glomerular podocyte hypertrophy. Additionally, high glucose also induced podocyte hypertrophy via activation of the IL-6/Gp130-JAK/STAT3 signal pathway.

Although the pathophysiology of podocyte hypertrophy in the initial stage of DN is still ambiguous, animal and human studies have established that glomerular podocyte hypertrophy was associated with the development of DN [9, 10]. Previous researches indicated that MAPK, TGF-*β*, and AngII had different effects on mesangial matrices and cells, leading to glomerular hypertrophy in the progress of DN. However, elaborate mechanisms underlying podocyte hypertrophy were less reported [11]. Romero et al. [12] concluded that AngII increased expressions of parathyroid hormone-related protein (PTHrP), TGF-*β*1, and cell cycle regulatory protein-p27Kip, which promoted the aggravation of podocyte hypertrophy in high glucose. The mammalian target of rapamycin (mTOR) signaling mainly consisted of mTORC1 and mTORC2. Several studies suggested that mTORC1 was closely associated with the activation of podocyte hypertrophy which was induced by high glucose [13]. In the early stage of diabetes, it was obviously found that high filtration of glomerular is accompanied with podocyte hypertrophy. In general, mature podocytes had to expand the size of themselves in order to compensate for glomerular dilation, which contributed to cover the denuded region of the GBM, because they were terminal-differentiated cells, which no longer are proliferated [14, 15]. Jo et al. [16] declared that interleukin 6 (IL-6) and its downstream cascade signaling proteins, such as Gp130 signal transducer and activator of transcription 3 (STAT3) signal transducer, were key regulators related to podocyte hypertrophy in a high-glucose environment. In general, hyperglycemia induced the overexpression of nuclear STAT3 via the activation of upstream signal transduction element Gp130, which is eventually leading to podocyte hypertrophy. Kim et al. [17] found that TCTP, as a mediating signal of cell growth, was overexpressed with high percentage in the glomeruli of diabetic mice and gave rise to podocyte hypertrophy. Fluorescent double-labeling method indicated that TCTP was mainly observed in podocytes. Studies showed that TCTP could activate the mTORC1 signal pathway and promote high expression of CKIs, which caused podocyte cycle arrest and hypertrophy. On the contrary, overexpression of mTORC1 and CKIs could be inhibited by gene knockout of TCTP, to make the podocyte bodies smaller. Meanwhile, in vitro experiments indicated that TCTP inhibitor could downregulate the expression of CKIs, ameliorating podocyte hypertrophy caused by high glucose. Kim et al. [18] showed that AngII could also upregulate protein expressions of kinases ERK1/2 and Akt/PKB, which contributed to podocyte hypertrophy. Hence, it has been shown that all ERK1/2, Akt/PKB, IL-6/JAK2/STAT3, and mTOR signal-pathway activities had important roles in podocyte hypertrophy (Figure 1()).

### 3.2. Podocyte Epithelial-Mesenchymal Transition

**Figure 2:**
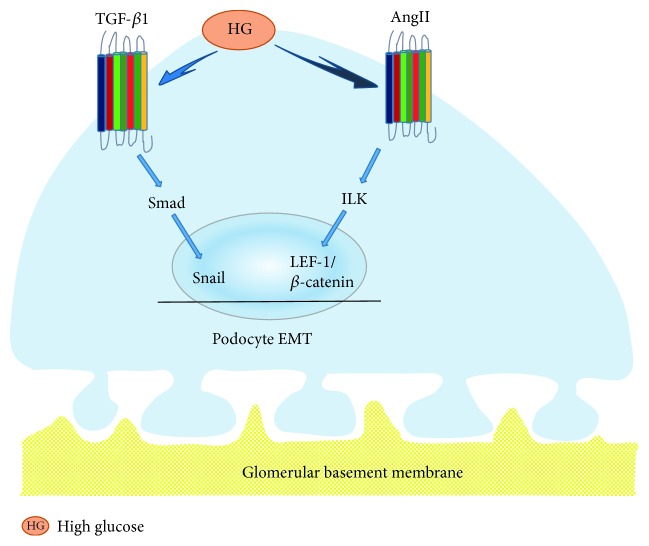
Podocyte EMT. In elevated glucose levels, the TGF-*β*1/Smad signal pathway resulted in increased protein expression of Snail in cultured podocytes, which induced podocyte EMT. Additionally, AngII promoted translocation of *β*-catenin/LEF-1 complexes into the nucleus through the enhancement of ILK in transitioning epithelia, where they downregulated CD2AP expression via promoting EMT transcription in podocytes.

Previous studies have shown a connection between podocyte apoptosis and proteinuria. However, more and more studies have demonstrated that normal epithelial cells showed phenotype conversion of a variety of nephropathies [19]. Cells lost their original features when the pathological process of EMT occurred, which induced disappeared cell contact, damaged cell polarity, and recaptured characteristics of the mesenchymal markers, such as vimentin, *α*-smooth muscle actin (*α*-SMA), and fibroblast-specific protein 1 (FSP1). FSP1 is one of the important members of calcium-binding protein S100 family and also a fibroblast-specific protein without epithelial cells [20]. Many studies showed that renal tubular epithelial cells and podocytes were activated after acute (48–72 h) exposure of cells to elevated glucose levels or other stimulations of diabetes, which resulted in less protein expressions of E-cadherin and ZO-1. Conversely, expressions of transdifferentiation proteins, such as *α*-SMA and vimentin, were immediately increased after these stimulation [21–23]. In the initial stage of STZ-induced diabetes, morphology of podocytes were damaged, accompanied with increased expression of the podocyte marker, nephrin protein, and a fall in the mesenchymal marker, desmin protein [24]. Yamaguchi et al. [25] found that FSP1-positive cells were significantly increased in urinary sediment and approximately attached to 86 percent of total podocytes in 109 type 2 diabetes patients. 43 of these patients with massive proteinuria in this study experienced kidney biopsy. The FSP1 positive cells selectively expressed Snail and ILK preferentially, which played pivotal roles in inducing EMT. Xing et al. [26] demonstrated that podocyte incubated in elevated glucose levels for 48 h could trigger activation of the PI3K/AKT pathway and elevate protein expressions of *α*-SMA and desmin. Whereas, protein expressions of podocalyxin and nephrin were suppressed. Functionally, it is apparently speculated that podocyte EMT may be tightly related to the PI3K/AKT signal pathway. Li et al. [27] found that the elevated glucose level upregulated protein expression of Snail and suppressed protein expressions of P-cadherin and nephrin in vitro. The change above decreased podocyte-related proteins of nephrin and ZO-1, elevated expressions of desmin, MMP9, and FSP1 in sequence. As shown in Figure 2, podocyte EMT widely participated in the early stage of podocyte deletion in diabetes mellitus via leading to podocyte detachment or podocyte apoptosis [28].

### 3.3. Podocyte Detachment

**Figure 3:**
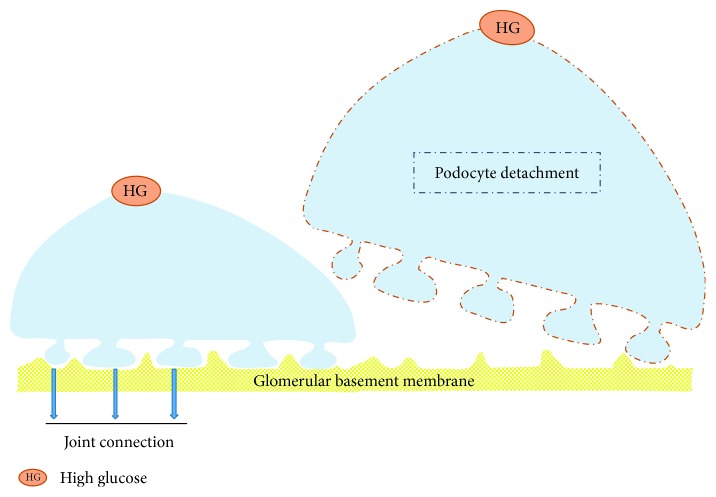
Podocyte detachment. Some podocytes became hypertrophic and stripped from the basement membrane in sequence, while the others tightly combined with basement membrane at the same stage of DN, which seems to be more related to podocyte EMT.

Podocytes and the glomerular basement membrane (GBM) are closely connected and then prevent the excretion of proteinuria via sustaining the glomerular filtration barrier. Researches showed that not only dead podocytes but also normal podocytes were found in the urinary sediment of patients with kidney disease. Furthermore, it had been concluded that podocytes could be cultured from urine of a healthy person [22]. One study displayed that urinary podocyte might be used as an earlier biomarker of DN than proteinuria albuminuria in respect of renal injury [29]. It was generally known that integrin *α*3*β*1 was an important receptor which could tightly connect podocyte with the GBM [30]. Jim et al. [31] drew a conclusion that the expressions of podocyte marker proteins in the diabetic kidney, such as synaptopodin, podocin, and nephrin, were significantly decreased, which could result in podocyte cytoskeleton disorder, damaged sufficient adhesion, and separation between podocyte and the basement membrane. Hyperglycemia could downregulate expression of integrin *α*3*β*1 in both human and rat, as well as trigger activation of integrin-linked kinase (ILK). In addition, recent researches suggested that *α*3*β*1 participated in the adhesion function of podocyte [32, 33]. Experimental researches on animals displayed that podocytes could break away from the glomerulus basement membrane in an artificial diabetic rat induced by streptozotocin (STZ) [34]. It was also suggested that both podocyte detachment and podocyte early changed in DN. However, whether podocyte detachment or podocyte hypertrophy appeared earlier was still difficult to distinguish [35]. It was obviously found that podocyte loss contributed to the development of DN. In the progression of disease, we found that in the same stage of DN, some podocytes became hypertrophy and detached from the basement membrane. Whereas, the others tightly combined with the basement membrane [36] (Figure 3()).

### 3.4. Podocyte Apoptosis

**Figure 4:**
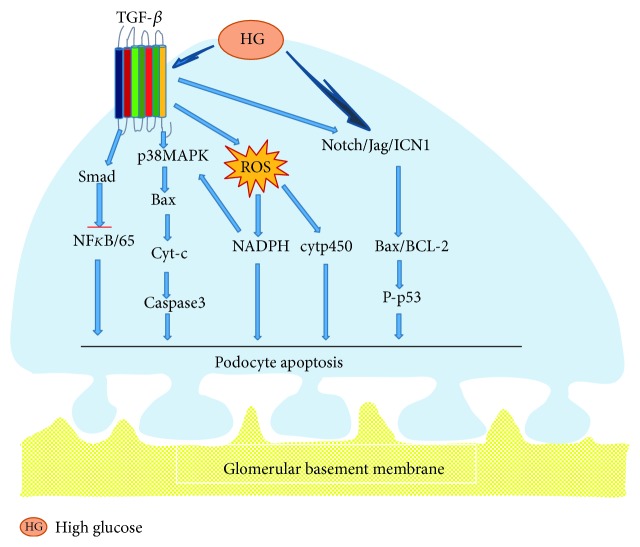
Podocyte detachment. In elevated glucose levels, TGF-*β* provoked p38 MAPK, Smad signal pathway and NADPH, and cytp450 to increase the protein expressions of Bax, cytochrome C, and caspase-3, which resulted from overproduction of ROS. All of the above processes could result in podocyte apoptosis. They also stimulated the Notch/Jag/ICN1 signal pathway, which activated the Bcl-2 and p53 apoptotic pathways and induced podocyte apoptosis.

Apoptosis pathway is involved in cellular growth and differentiation in many diseases, such as DN and IgA nephropathy. There are some evidences that podocyte apoptosis played a role in reduction in density and number of glomerular. Susztak et al. [37] found that mitochondria could activate nicotinamide adenine dinucleotide phosphate (NADPH) oxidase and reactive oxygen species (ROS) in high glucose, improve the expressions of p38 protein kinase and caspase 3 at the same time, then led to podocyte apoptosis, and produced much proteinuria. Glycosylation end products activated transcription factor FOXO4, which also induced podocyte apoptosis via p38 protein kinase signaling pathways [38]. In addition, the cytochrome P450 family raised hydroxyl and reduced coenzyme II twenty-four carbon olefine acid oxidase in high glucose, which increased the active oxygen class produces and prompting podocyte apoptosis [39]. Notch1 signal-dependent activation of p53 is a new podocyte apoptosis pathway [40]. A recent study found that podocyte apoptosis was closely related to the expression level of Notch, which then induced proteinuria and glomerular sclerosis. The activation of Notch signaling pathway may be included in DN all-acquired common mechanism of kidney disease [41]. Experiment with STZ-induced diabetes mouse displayed that the expressions of Jag, Notch, and ICN1 were increased immediately and the downstream component, such as Hes1 and Hey1, were activated. It is also shown that the expressions of Bcl-2 and p53 were provoked, and the process eventually induced podocyte apoptosis [42]. Under normal circumstances, apoptosis-promoting and antiapoptosis signaling pathways coexisted in a same condition which maintained dynamic balance and guaranteed the stability environment. In general, phosphatidylinositol-3 kinase/protein kinase in podocyte plays a crucial role in inhibition of podocyte apoptosis signaling pathways. The experiment also suggested that protein kinase phosphorylation reduced in db/db mice, which may be an important inducement for podocyte apoptosis [43]. Liu et al. [44] found that podocyte apoptosis was associated with the disorder of cytoskeleton. Nestin is a VI intermediate filament protein related cell cytoskeleton which expressed in podocyte. The protein expression of nestin reduced in high glucose, however, podocyte apoptosis rate increased. TGF-*β*1 could directly activate Smad7, which inhibit the activity of NF-kB and resulted in podocyte apoptosis. It also could provoke p38 MAP kinase, enhance the protein expressions of Bax and produce cytochrome C, which activated caspase-3 apoptosis pathway in sequence [45]. Liu et al. [46] demonstrated that metadherin was a potent modulator of podocyte apoptosis, which represented the target of miR-30 miRNAs, facilitating podocyte apoptosis via activating the HG-induced p38 MAPK-dependent pathway. Yao et al. [47] concluded that AS-IV inhibited podocyte apoptosis induced by high glucose, which reduced the expressions of TRPC6 and impaired the crosstalk of intracellular Ca2+ in podocytes. More and more evidences [48] indicated that AS-IV could protect the kidney from DN, including reduced podocyte damages and suppressed podocyte apoptosis through antioxidative stress and anti-inflammatory signaling pathways (Figure 4()).

## 4. Main Signaling Pathways of Podocyte Injury Mechanism in DN

Many studies [49] suggested that DN podocyte injury was induced by the association of multiple factors, including mechanical stress, inflammatory reaction, oxidative stress, TGF-*β*1 induction, renin angiotensin aldosterone system (RAAS) activation, and AGEs accumulation. And there are lots of signaling pathways involved in the regulation mTOR signaling pathways mediated by autophagy, adenosine monophosphate-activated kinase (AMPK) signaling pathway, Wnt/*β*-catenin signaling pathway, and so on.

### 4.1. Adenosine Monophosphate-Activated Kinase Signaling Pathways (AMPK) in DN

AMPK is not only a serine protein kinase [50] playing a vital role in cells and tissues metabolisms of the diabetes progression but also one of the important metabolic emergency protein kinases. AMPK pathway is an autophagy-related signaling pathway composed of subunit heterologous proteins *α*, *β*, and *γ*, which was activated as lack of energy in cells [51]. In the activation process, it could combine calmodulin-dependent kinase *β* (CaMKK) and transform-activated kinase 1 (TAK-l). Then, it also effectively mediated intracellular calcium concentration and triggered the activation of the AMPK pathway to induce autophagy [52]. Conversely, AMPK could inhibit mTORCl activity and induce autophagy through TSCl/2-Rheb signaling pathways and/or phosphorus acidification of raptor-related regulatory protein [53]. Meanwhile, AMPK also directly launch the phosphorylation of Ulkl/2 and induction of autophagy [54]. In addition, Sharma et al. [55] reported that adiponectin attenuated the induction of oxidative stress, reduced the synthesis of NADPH in podocyte, and simultaneously reduced albuminuria excretion in adiponectin-knockout mouse which could activate the AMPK pathway.

### 4.2. Wnt/*β*-Catenin Signaling Pathways in DN

Wnt protein is one kind of secreted glycoprotein, containing a signal peptide and 23 or 24 conserved cysteine residues. It was activated via binding to ligand proteins and the Frizzled protein family. Wnts triggered a cascade of downstream reaction protein including axin, Disheveled, adenomatous polyposis coli (APC), and glycogen synthase kinase- (GSK-) 3*β*, which gave rise to phosphorylation of *β*-catenin in nuclei [56]. Researches showed that Wnt signaling pathway had effects on the differentiation, hyperplasia, maturation, and viability of cells [57]. It had been declared to be induced in DN, which played a crucial role in apoptosis and EMT formation of mesangial cells, podocyte, and tubular cells [58]. However, Dickkopf-related protein 1 (DKK1) is a secreted protein consisted of two cysteine abundant regions, which could reduce podocyte injury, decrease albuminuria, and protect the kidney by specific blocking Wnt/*β*-catenin signal pathways [59, 60]. Liu et al. [61] found that curcumin could prevent glomerular podocyte injury by inhibiting activated Wnt family members and *β*-catenin downstream effectors in obesity-related glomerular disease model. Zhang et al. [62, 63] demonstrated that ubiquitin carboxy-terminal hydrolase-1 (UCH-L1) is abnormally expressed in injury podocytes, especially in immune-mediated disease. They also proved that the Wnt/*β*-catenin signal pathway is promptly activated in podocyte, coinciding with overexpression of UCH-L1 induced by high glucose meanwhile [64]. Li et al. [65] announced that podocyte incubated in high glucose underwent injury, which attributed to the upregulation of transient receptor potential cation channel 6 (TRPC6) protein triggered by the classic Wnt/*β*-catenin pathway.

### 4.3. Endoplasmic Reticulum Stress-Related Signaling Pathways in DN

Recent studies suggested that endoplasmic reticulum stress was closely relevant to the injury of podocytes, endothelial cells, and mesangial cells in DN. It could induce glomerular obstacle of podocyte structure and function, participate in a variety of kidney diseases, and also lead to glomerular sclerosis [66]. Continuous endoplasmic reticulum stress had effects on the function of endoplasmic reticulum and could launch apoptosis signaling pathways which were mediated by endoplasmic reticulum stress at the same time and then activated the downstream apoptotic signaling molecules [67]. In patients with diabetes, hyperglycemia can motivate endoplasmic reticulum stress through a variety of ways, then cause cellular damage [68]. As important factors of DN, advanced glycation end products (AGE) could upregulate the protein expressions of glucose-regulated protein 78 (GRP78) and induce endoplasmic reticulum stress depending on its dosage and time, eventually inducing apoptosis of podocyte [69]. In addition, high glucose and free fatty acids could induce endoplasmic reticulum stress as well as the occurrence of apoptosis in podocyte of rats, which could be inhibited via exogenous endoplasmic reticulum molecular chaperone [70]. Endoplasmic reticulum stress may aggravate podocyte dysfunction in the early stage of DN [71]. The relationship between endoplasmic reticulum stress and podocyte injury could be summarized as follows: both hyperglycemia and AGE could initiate endoplasmic reticulum stress and activate mTORC-1 protein. Furthermore, continuous injury will contribute to podocyte apoptosis by the caspase-12 pathway, while AGE could act on collagen type IV, leading to podocytes loss or dysfunction [15, 72].

### 4.4. mTOR Signaling Pathways Mediated by Autophagy in DN

Studies have shown that intervention in the activity of the mTOR signaling pathway is likely to aggravate podocyte injury in DN renal tissue [73]. Activated podocytes in mTORC1 could result in dislocation of nephrin protein, ZO-1 (skeleton protein) disorders, and podocyte EMT, which could eventually lead to podocyte detachment, foot process fusion and disappearance, and other podocyte injuries in Inoki knockout PcKO Tsc1 mouse. In conclusion, mTORC1 activity has a key regulatory role in podocyte injury on the DN model of rats [15]. In addition, many pharmacological studies of mTOR inhibitors have further elucidated the importance of mTOR in mediating DN podocyte injury from another perspective such as rapamycin-protected podocytes [15, 74]. Recent researches have indicated that autophagy, a protective mechanism of podocyte, was used to against damage in a variety of pathological factors. On the contrary, autophagy defects not only led to podocyte injury and proteinuria but also aggravated glomerular sclerosis [54, 75, 76]. Rapamycin, as the mTOR inhibitor, acted on the autophagy pathway and protected the podocyte [55]. In conclusion, there were two kinds of defense mechanism that the autophagy/mTOR signal pathway is using to protect podocytes from further injury. The pathway of mTORC1 is activated and the protective autophagy is inhibited when podocyte is in a high-glucose environment. Specifically, mTORC1 inhibited the autophagosome by initiating the activity of UNC-51-like kinase 1 (ULK1) [13, 77]. In addition, rapamycin could upregulate the protein expression of 1A/1B light chain 3 (LC3) in vitro and improved podocyte autophagy disorders in sequence [78].

### 4.5. Rho/ROCK Signaling Pathway in DN

The Rho family is mainly composed of RhoA, Rac1, and Cdc42. They were the vital mediators of the actin cytoskeleton protein structure [79]. RhoA/ROCK pathway was the important process in the progression of DN and could induce downstream signaling element cell apoptosis, migration, and differentiation [80]. Immoderate activities of Rac1, a key element in the Rho GTPases family, could cause macroalbuminuria quickly with focal foot process effacement, indicating podocyte apoptosis and slit diaphragm protein expression reductions in high glucose [81, 82]. Wang et al. [83] found that Drp1 at serine 600, as a substrate of Rho signal pathway, not only initiated mitochondrial ROS and podocyte apoptosis in high glucose but also was phosphorylated in ROCK1 knock-out mouse. RhoA played an important role in glomerular filtration barrier integrity, and its overexpression could damage the structure and function of the barrier [84, 85]. Phosphatase and tensin homolog (PTEN) could inhibit the activation of the RhoA/Rac1/Cdc42 signaling pathway, which contributed to reverse the cytoskeletal rebuilding and prevent the development of DN [86, 87]. Previous studies demonstrated that Rho-GTPase family elements were probably activated to induce the downstream cascade reaction when they were exposed in the environment of diabetics, such as AGEs, hyperglycemia, oxidized LDL, and ROS [88, 89]. Xie et al. [90] suggested that Berberine not only inhibited RhoA/ROCK to improve DN but also regulated Rho GTPases to reduce oxidative stress.

## 5. Conclusion

**Figure 5:**
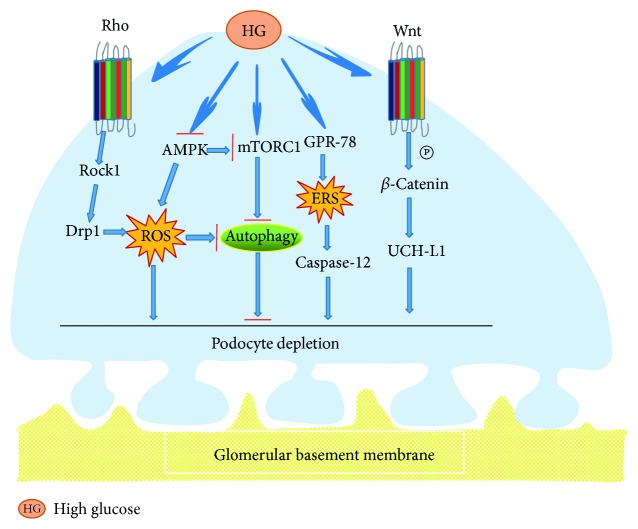
Signaling pathways underlying podocyte injury in DN. Hyperglycemia induced podocyte injury through the Rho/ROCK1 and AMPK signal pathways. Part elements of the signal pathway initiated autophagy that protects from podocyte injury through ROS or mTORC1 participation in the active process. Additionally, GPR-78 activated ERS to induce podocyte apoptosis based on the caspase-12 signal pathway, and the Wnt/*β*-catenin signal pathway may have similar changes in podocyte injury, which could induce podocyte depletion via upregulating expression of UCH-L1 in high glucose.

Podocyte injury is an important factor in DN progression. Several studies implied that the process of albuminuria development in DN was complicated, which presumably included four phases in sequence as follows: podocyte hypertrophy, podocyte EMT, podocyte detachment, and podocyte apoptosis, serving as the warning mark of GFR in DN [91]. More and more signal pathways which induced podocyte injury have been discovered [92], such as Wnt/*β*-catenin signaling pathways, Rho-GTPase signaling pathways, mTOR signaling pathways, and endoplasmic reticulum stress-related signaling pathways (Figure 5). People have understood the multiple pathogenesis mechanisms of podocyte injury in DN, but the complex clinical manifestations of that tell us that there is still potential knowledge required to study and discuss.
